# Signs of Heart Disease

**Published:** 1855-05

**Authors:** 


Signs of Heart Disease.—

1. That cases of valvular affection may be divided into two classes, in one of which the disease has been produced by inflammation, while, in the other, it appears to arise independently of this condition.

2. That in the first class of cases, a period arrives in which, although the disease is progressive, there is no evidence of its being of an inflammatory nature.

3. That hence it is generally improper to persist in an antiphlogistic treatment of valvular disease beyond a certain period of time.

4. That the determination of the actual seat and nature of a valvular disease is of less importance than that of the vital and mechanical state of the heart.

5. That a permanently patent state of the orifices is the ordinary result of all valvular diseases. This condition may or may not be attended with contraction, or the orifices may be dilated.

6. That the period when inadequacy of the valves supervenes, varies greatly in different cases.

7. That hence, two series of phenomena may occur; in the first we have the signs of disorganization without inadequacy; in the second those of inadequacy are added.

8. That the distinctness of valvular murmur cannot be taken as being proportionate to the amount of disease.

9. That a complete cessation of murmur may coincide with the advance of the disease.

10. That the cessation of murmur, under these circumstances, has been only observed in connexion with contraction of the orifice; it has not been observed in cases of free regurgitation.

11. That absence of murmur does not necessarily imply absence of valvular disease, especially if there be symptoms of disease of the cavities.

12. That the number of cases in which we are warranted in making a special diagnosis of valvular disease is small.

13. That the number of pathological conditions competent to cause such changes in the valves as will produce murmur is very great.

14. That in the earlier periods of valvular disease, murmur may not occur, although the disease be progressive.

15. That even in chronic cases, the development of murmur may be sudden.

16. That the disorganizing process may advance with great rapidity, or with slowness, and that, in some cases, it appears to be really arrested.

17. That the irregular action of the heart is much more related to the state of the cavities than to that of the valves.

18. That we may observe the sudden development of the symptoms as well as of the physical signs of chronic disease of the heart.

19. That three conditions of the heart, considered in its vital relations, may accompany or follow valvular disease:—

1.—Increased force of the heart.

2.—Diminished force, with rapidity and irregularity of action.

3.—Diminished force, with remarkable slowness and comparative regularity of action.

20. That the law which regulates the production of the alteration of the cavities, which follows on valvular obstruction, with or without inadequacy, is still undetermined.

21. That considering the rarity of organic change in the valves at the right side of the heart, and the difficulty or impossibility of their special diagnosis, we may, in a practical point of view, limit our considerations to the diseases of the mitral and aortic valves.

22. That in the diseased and permanently patent condition of the valves of the pulmonary artery, a double murmur at the base of the heart, not propagated into the aorta, and not attended with general arterial throbbing, has been observed.

23. That in most cases of organic disease of the valves at the right side of the heart there is either an open foramen ovale, or a deficient ventricular septum.

24. That the most frequent result of disease of the right auriculo-ventricular valves is but the exaggeration of their natural insufficiency.

25. That we cannot by the ordinary acoustic tactile signs determine the existence of dilatation of the right auriculo-ventricular orifice.

26. That reflux pulsations in the veins of the neck, and occasionally in those of the upper extremities, indicate regurgitation into the right auricle.

27. That hence they may be taken as indicating the insufficiency of the valves, and may have, as their remote cause, morbid conditions of the pulmonary artery, the lung, or the left side of the heart

28. That of these different lesions the most frequent is contraction of the mitral orifice.

29. That the venous pulse thus produced may be permanently present, or only developed during an attack of cardiac asthma.

30. That the pulsations in the jugular veins are synchronous and isochronous with the ventricular systole.

31. That we must not depend on any acoustic character of the murmur, nor even on its exact seat, for the diagnosis of valvular disease. It is requisite to combine with these considerations those of the history and symptoms of the case, as well as those which have reference to the state of the pulse, the force of the heart, and the condition of the lung and liver.

32. That all diagnostics depending solely on the tone, character, and seat of murmur, are more or less doubtful.

33. That although by acoustic signs we may often determine the insufficiency of a valve, yet there are no means by which, from the stethescope alone, we can declare the cause of that insufficiency.

34. That the diagnostics between the contraction and dilatation of any of the orifices, founded on acoustic phenomena, are to be rejected.

35. That organic and anæmic murmurs may co exist.

36. That there are no distinctive symptoms of disease of the mitral valves, when it is uncomplicated with alteration in the vital or mechanical state of the cavities.

37. That its principal physical indication is a murmur which is systolic, but not propagated into the arteries, and loudest towards the apex and to the left side. This may or may not be attended with fremitus.

38. That the most common result of contraction of the mitral opening is pulmonary congestion, with enlargement of the right cavities of the heart.

39. That under these circumstances, from the preponderance of the right ventricle, a globular form of the heart may be produced.

40. But a globular form of the heart may exist with a dilated mitral opening, attended with enlargement of the left ventricle, while the right remains unaffected.

41. That the combination of a contracted state of the mitral opening, with permanent patency of the aortic valves, is of frequent occurrenee.

42. That under these circumstances, we may occasionally observe both the aortic and the mitral murmurs.

43. But that the absence of a mitral murmur, in a case of permanent patency of the aortic valves, does not necessarily imply that the auriculo-ventricular opening is free from disease.

44. That in cases of mitral contraction moveable coagula may be formed in the left auricle, which may, by occlusion of the opening, become a cause of sudden death.

45. That with the progress of contraction, the mitral murmur may gradually subside, and ultimately become extinct, so that with the increase of disease, we may have decrease and cessation of murmur.

46. That this cessation of murmur may coincide with a permanently patent though contracted opening.

47. That inasmuch as most cases of mitral murmur are systolic, they are to be held as regurgitant. We cannot by acoustic signs, distinguish between the direct constrictive and the regurgitant murmur.

48. That the interscapular murmur may attend constriction or dilatation of the mitral opening, but appears more allied to the latter than to the former condition.

49. That the interscapular murmur may be consequent on a recent and acute disease of the heart.

50. That the existence of a presystolic murmur, which theoretically should imply that it attended the passage of blood from the auricle into the ventricle, does not justify the diagnosis of absence of regurgitation through the mitral orifice.—*Stokes on Diseases of the Heart and Aorta.*

